# Synthesis of *All*-*Z*-1,6,9,12,15-Octadecapenten-3-one, A Vinyl Ketone Polyunsaturated Marine Natural Product Isolated from *Callysponga* sp.

**DOI:** 10.3390/molecules19033804

**Published:** 2014-03-24

**Authors:** Anne Marie Langseter, Yngve Stenstrøm, Lars Skattebøl

**Affiliations:** 1 Department of Chemistry, Biotechnology and Food Science, Norwegian University of Life Sciences, P.O.Box 5003, NO-1432 Ås, Norway; 2 Department of Chemistry, University of Oslo, P.O. Box 1033, Blindern, NO-0315 Oslo, Norway

**Keywords:** *Callyspongia* sp, polyunsaturated fatty acid, EPA, DHA, iodolactonization, synthesis, marine sponge metabolite

## Abstract

The synthesis of the marine natural product 1,6*Z*,9*Z*,12*Z*,15*Z*-octadecapentaen-3-one (**1**) has been achieved by two different routes starting from the ethyl esters of eicosapentaenoic acid (EPA) and docosahexaenoic acid (DHA), respectively. Using EPA ethyl ester as starting material the polyunsaturated vinyl ketone lipid **1** was obtained in 17% overall yield.

## 1. Introduction

Over the years numerous polyunsaturated lipids have been isolated and characterized as secondary metabolites from marine sources [1]. Several possess interesting biological properties and are targets for total syntheses. Some years ago 1,6*Z*,9*Z*,12*Z*,15*Z*-octadecapentaen-3-one (**1**) was isolated from an Australian marine sponge *Callyspongia* sp by Urban and Capon [2]. Few fatty acid derived natural products with a vinyl ketone moiety have been described. No biological activity was reported for compound **1** and to best of our knowledge it has not been synthesized yet.

The vinyl ketone **1** attracted our interest as part of an ongoing project utilizing the polyunsaturated fatty acids eicosapentaenoic acid (EPA) and docosahexaenoic acid (DHA) as starting materials for the syntheses of compounds containing several methylene interrupted *Z* double bonds [3,4,5,6,7,8,9,10,11,12,13,14,15,16,17]. A key reaction in this respect is the oxidative degradation of EPA and DHA to aldehydes by way of the corresponding iodolactones. In the present work a simplified iodolactonisation procedure for EPA and DHA is presented as part of the synthesis of vinyl ketone **1**.

## 2. Results and Discussion

**Scheme 1:**
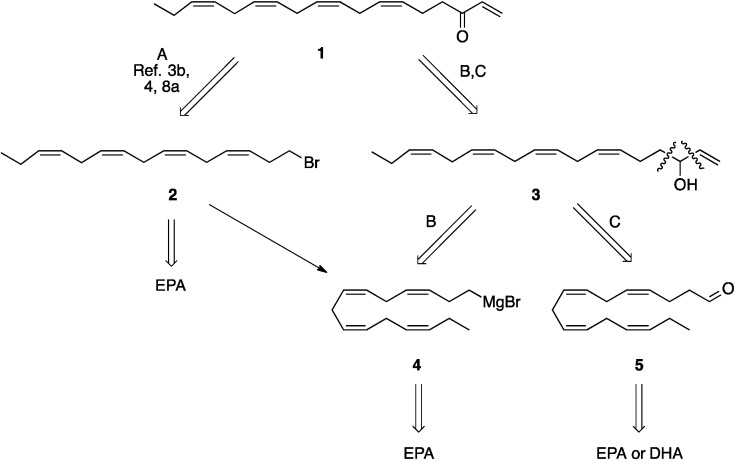
Retrosynthetic analysis of the target molecule **1**.

With the use of EPA and DHA in mind three different routes to the target molecule **1** became apparent, as outlined in Scheme 1. Routes A and B proceed through the bromide **2** which is available from EPA ethyl ester by modification of a literature procedure [4,17,18] to improve the yields. The vinyl alcohol **3** is an intermediate in both routes B and C.

Route A seemed promising at first. Katritzky and coworkers have published an elegant one step method for the generation of vinyl ketones by the reaction of the propenoyl anion equivalent *N*-(α-ethoxyallyl)benzotriazole with a bromide [19]. Unfortunately, reaction of the bromide **2** with the propenoyl anion equivalent under the reported conditions gave no vinyl ketone and elimination products were the only detectable compounds according to NMR.

**Scheme 2:**
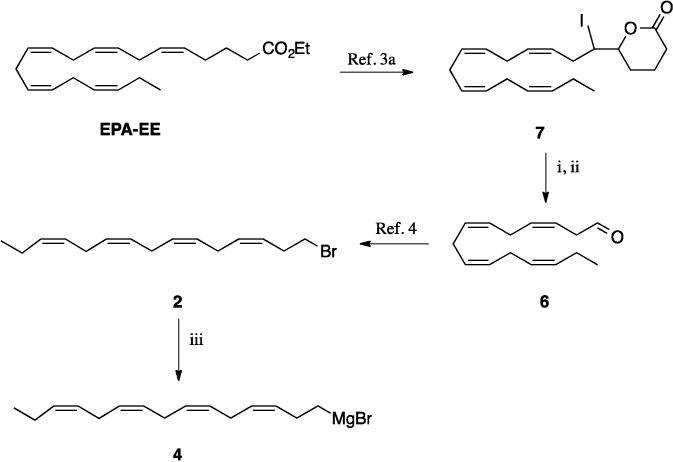
Synthesis of the key intermediate bromide **2**.

**Scheme 3:**
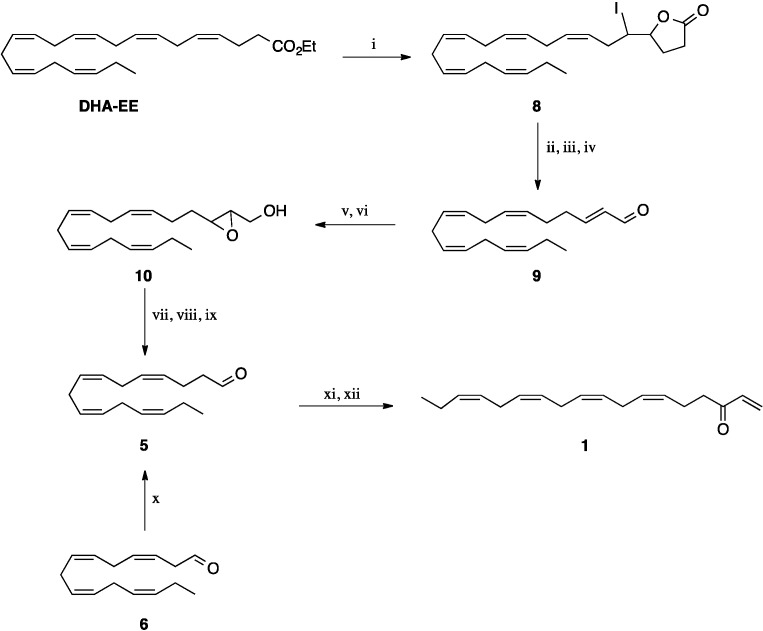
Alternative synthesis of **1** starting with EPA.

We then attempted route B as depicted in Scheme 2. However, transformation of the bromide **2** to the Grignard reagent **4** was accompanied by a high degree of Wurtz coupling; consequently, the reaction with acrolein resulted in a very poor yield of the vinyl alcohol **3**. Difficulties with an efficient formation of this Grignard reagent have been observed recently, and attempts to prepare the lithium analogue of **4** by lithium exchange on the corresponding iodide resulted in significant isomerisation of the double bonds [4]. Discouraged by this result we turned our attention to route C that required the C-16 aldehyde **5**, available from EPA and DHA as outlined in Schemes 1 and 3. Starting from DHA using the modified iodolactonisation procedure and oxidative cleavage, followed by the DBU-induced isomerisation of the β,γ-double bond, the conjugated aldehyde **9** was obtained in good overall yield. The corresponding alcohol derived from compound **9** underwent Sharpless epoxidation. Protection of the epoxyalcohol **10**, epoxide opening to the diol and oxidative cleavage of this afforded the aldehyde **5** [20].

*Reagents and conditions:* (i) 5% LiOH in MeOH-H_2_O (19:1), reflux, 6 h, 99%; (ii) (a) 5% LiOH in MeOH-H_2_O (19:1), 0 °C, 30 min.; (b) sat. aq. citric acid to pH 4 then NaIO_4_ rt, 1 h, 80%; (iii) Mg, THF.

*Reagents and conditions:* (i) (a) LiOH, EtOH/H_2_O (1:1), rt, 4 h; (b) HI, KHCO_3_, I_2_, 0–4 °C, 18 h, 97%; (ii) (a) K_2_CO_3_, dry MeOH, overnight rt; (b) 5% LiOH in MeOH-H_2_O (19:1), reflux, 6 h, 85%; (iii) (a) 5% LiOH in MeOH-H_2_O (19:1), 0 °C, 30 min; (b) sat. aq. citric acid to pH 4 then NaIO_4_, rt, 1 h; (iv) DBU, 74% from the diol acid (two steps); (v) NaBH_4_ (See ref. 17, 80%); (vi) Ti(*i*-OPr)_4_, *t*-BuOOH, DCM, −20 °C, 64%; (vii) MsCl, 2,6-lutidine, 68%; (viii) 10% HClO_4_, THF, 49%; (ix) NaIO_4_, rt, 1 h, 50%; (x) (a) MeOCH_2_PPh_3_Cl, *t*-BuOK, overnight; (b) HCO_2_H-H_2_O, overnight, 38%; (xi) CH_2_=CHMgBr, THF, 0 °C, 1 h, 63%; (xii) DMP, DCM, 89%.

This route was quite lengthy so we switched to an approach involving chain elongation of aldehyde **6** by a Wittig reaction as depicted in Scheme 3.

Treatment of the aldehyde with the ylide derived from methoxymethyltriphenylphosphonium chloride and potassium *t*-butoxide followed by hydrolysis of the resulting vinyl ether gave the aldehyde **5**. Finally, reaction of **5** with vinylmagnesium bromide followed by oxidation of the allylic alcohol using the Dess-Martin periodinane procedure gave **1** in 21% yield overall from **5**. The spectral data compared well with those reported for the natural compound [2].

## 3. Experimental

### General

All reactions were performed under nitrogen. EPA ethyl ester was obtained from Pronova Biopharma, Sandefjord, Norway. All other reagents were used as purchased. The NMR spectra were recorded on a Varian Gemini spectrometer. MS (EI) spectra were recorded on an Autospec Ultima instrument and are presented as *m/z* (% relative intensities). HRMS were recorded on the same instrument. IR spectra were obtained on a reflectance cell on a Perkin Elmer FT-IR instrument. The syntheses of some of the compounds have been previously published. When included here, modifications and/or improvements of yields have been obtained.

*5,6-Dihydroxy-(8Z,11Z,14Z,17Z)-icosatetraenoic acid* (**11**). A solution of the iodolactone (**7**) [3] (12.09 g, 28.2 mmol) and 5% LiOH^.^H_2_O in MeOH-H_2_O (19:1, 120 mL) was refluxed for 6 h. Water (120 mL) was added and most of the methanol was removed under reduced pressure. The reaction mixture was cooled (ice bath) and acidified with dilute HCl. Solid NaCl was added to saturation and the reaction mixture extracted with EtOAc (3 × 50 mL). The organic extracts was washed with brine (2 × 50 mL), dried (Na_2_SO_4_) and solvent was removed under reduced pressure to obtain **11** (9.46 g; 99%) as a yellow oil. Occasionally small amounts of the corresponding hydoxylactone were seen in the NMR spectra. Spectral data were in agreement with those previously reported [21].

*3Z,6Z,9Z,12Z-pentadecatetraenal* (**6**). A mixture of the dihydroxy acid **11** (9.55 g, 28.3 mmol) and 5% LiOH**^.^**H_2_O in MeOH-H_2_O (19:1) (90 mL) was cooled on an ice-bath and left stirring for 30 min. before water (90 mL) was added. A solution of saturated citric acid was added until pH 4 was attained in the reaction mixture. Solid NaIO_4_ (7.5 g, 35 mmol) was added in one portion. The reaction mixture was left stirring at room temperature for 1 h. Solid NaCl was added to saturation and the product was extracted with hexane (3 × 50 mL). The extract was washed with brine (2 × 50 mL) and dried (MgSO_4_). Evaporation of the solvent under reduced pressure gave the unstable aldehyde **6** (4.88 g; 80%) as a colorless oil. Spectral data were in agreement with those previously reported [17].

*Dihydro-5-((3Z,6Z,9Z,12Z,15Z)-1-iodooctadecapentaenyl)furan-2(3H)-one* (**8**). A mixture of DHA ethyl ester (10.02 g, 28 mmol) and LiOH**^.^**H_2_O (5.8 g, 140 mmol) in EtOH-H_2_O (1:1) (60 mL) was left stirring until all the DHA ethyl ester was converted (TLC, CH_2_Cl_2_). Water (90 mL) were added, the reaction flask was covered with aluminium-foil and cooled to 0 °C. Hydrogen iodide (57%; 20 mL) was added to the reaction mixture, followed successively by saturated KHCO_3_ (10 mL) and dropwise addition of a solution of I_2_ (21.32 g, 84 mmol) in EtOH (70 mL). The mixture was left stirring at 0–4 °C in the dark for 18 h. The reaction was quenched by adding a saturated aq. solution of Na_2_S_2_O_3 _(100 mL). Solid NaCl was added to saturation and the product extracted with hexane (3 × 50 mL). The extract was washed with brine (2 × 50 mL), dried (Na_2_SO_4_) and evaporated under reduced pressure to give **8** (12.3 g; 97%) as pale yellow oil. Spectral data were in agreement with those previously reported [17].

*4,5-dihydroxydocosa-(7Z,10Z,13Z,16Z,19Z)-pentaenoic acid* (**12**). A solution of the iodolactone **8** (9.53 g, 21 mmol) in dry MeOH (110 mL) was cooled to 0 °C and K_2_CO_3_ (5.8 g, 40 mmol) was added. The mixture was left stirring overnight at room temperature. Water (12 mL) was added followed by a solution of 5% LiOH**^.^**H_2_O in MeOH-H_2_O (19:1) (90 mL). The mixture was refluxed for 4 h, cooled in an ice bath and acidified with dilute HCl. Solid NaCl was added to saturation and the product extracted with EtOAc (3 × 50 mL). The extract was washed with brine (2 × 50 mL) and dried (Na_2_SO_4_). Evaporation of the solvent under reduced pressure gave **12** (6.46 g; 85%) as an oil. Spectral data were in accord with the literature [21]. Small amounts of the corresponding hydroxylactone were inevitably present as shown by the NMR spectra [18,22].

*2E,6Z,9Z,12Z,15Z-octadecapentaenal* (**9**). A mixture of the crude dihydroxy acid **12** (6.46 g, 18 mmol) and 5% LiOH**^.^**H_2_O in MeOH-H_2_O (19:1) (60 mL) was cooled in ice and left stirring for 30 min. Water (60 mL) was added followed by a saturated solution of citric acid until pH 4 was attained. Solid NaIO_4_ (5.56 g, 26 mmol) was added in one portion and the reaction mixture left stirring at room temperature for 1 h. Solid NaCl was added to saturation and the reaction mixture was extracted with hexane (3 × 50 mL) The extract was washed with brine (2 × 50 mL) and dried (Na_2_SO_4_). Evaporation under reduced pressure left a residue that was dissolved in ether (125 mL) and DBU (1 mL) was added. After stirring for 30 min, the organic phase was washed with water to neutral pH, then with brine (2 × 50 mL) and dried (Na_2_SO_4_). The solvent was removed under reduced pressure furnishing the aldehyde **9** (3.44 g; 74%) as a yellow oil. Spectral data were in agreement with those previously reported [17].

*2E,6Z,9Z,12Z,15Z-octadecapentaen-1-ol* (**13**) was prepared by reduction of **9** with NaBH_4_ according to the literature [20].

*(3-((3Z,6Z,9Z,12Z)-pentadecatetraenyl)oxiran-2-yl)methanol* (**10**). A solution of the alcohol **13** (2.68 g, 10 mmol) in CH_2_Cl_2_ (5 mL) was added to solution of Ti(*O*-*i*-Pr)_4_ (3.55 mL, 12 mmol,) in CH_2_Cl_2 _(15 mL) precooled to −25 °C. The solution was allowed to stir for 20 min at −25 °C before *t*-BuOOH (8.72 mL, 30 mmol) was added. The reaction was left stirring overnight and quenched with the addition of 10 % aq. tartaric acid (5 mL) at 0 °C. After stirring for 30 min the solution was filtered trough a short pad of Celite. The reaction mixture was extracted with CHCl_3_ (3 × 25 mL). The organic extracts were washed with water (2 × 25 mL) and dried (Na_2_SO_4_). The residue was passed through a short pad of silica gel via reduced pressure eluting the epoxy alcohol with EtOAc. The epoxy alcohol **10** (1.74 g; 64%) was obtained as a pale yellow oil. The spectral data for **10** were in agreement with those previously reported [20].

*(3-((3Z,6Z,9Z,12Z)-pentadecatetraenyl)oxiran-2-yl)methyl methanesulfonate* (**14**). To an ice cooled solution of the epoxyalcohol **10** (1.71 g, 6 mmol) and 2,6-lutidine (2.1 mL, 18 mmol) in dry CH_2_Cl_2_(30 mL) MsCl (1.4 mL, 18 mmol) was added with stirring. The reaction mixture was left stirring for 2 h at room temperature. Brine was added and most of the CH_2_Cl_2_ was removed by evaporation under reduced pressure. The residue was extracted with EtOAc (3 × 25 mL). The extract was washed with water (2 × 25 mL), sat. NaHCO_3_ (2 × 25 mL), brine (2 × 25 mL) and dried (Na_2_SO_4_). Evaporation of the solvent under reduced pressure gave a residue that was passed through a short pad of SiO_2_ and K_2_CO_3_ with CH_2_Cl_2_ as eluent to give **14** (1.43 g; 68%) as a pale yellow oil. ^1^H-NMR (300 MHz, CDCl_3_) δ 5.23ߝ5.47 (m, 8H), 4.46 (dd, *J* = 3 Hz, *J* = 12 Hz, 1H), 4.08 (dd, *J* = 6 Hz, *J* = 12 Hz, 1H), 3.05 (s, 3H), 2.91(m, 1H), 2.73-2.86 (m, 7H), 2.17–2.26 (q, *J* = 6 Hz 2H), 2.06 (p, *J* = 9 Hz, 2H), 1.55–1.76 (m, 2H), 0.95 (t, *J* = 9 Hz, 3H). ^13^C-NMR (75 MHz, CDCl_3_) δ 132.29 (CH), 129.42 (CH), 128.84 (CH), 128.60 (CH), 128.43 (CH), 128.10 (CH), 128.00 (CH), 127.19 (CH), 70.06 (CH_2_), 56.36 (CH), 55.20 (CH), 38.07 (CH_3_), 31.52 (CH_2_), 25.84 (CH_2_), 25.80 (CH_2_), 25.75 (CH_2_), 23.78 (CH_2_), 20.78 (CH_2_), 14.52 (CH_3_). IR: 3011, 1652 cm^−1^. HRMS(EI). Calculated for C_19_H_30_O_4_S: 354.1865; Found 354.1896.

*(6Z,9Z,12Z,15Z)-2,3-dihydroxyoctadeca-6,9,12,15-tetraenyl methanesulfonate* (**15**). To a solution of the mesylate **14** (1.34 g, 3.8 mmol) in THF (30 mL) a 10% aqueous solution of HClO_4_ (24 mL) was added dropwise. The mixture was left stirring overnight at room temperature and quenched with a pH 7 phosphate buffer (1 M, 100 mL). The reaction mixture was saturated with solid NaCl and extracted with EtOAc (3 × 25 mL). The combined organic layers was washed with brine (2 × 25 mL) and dried (MgSO_4_). The residue obtained by evaporation of the solvent under reduced pressure was purified by flash chromatography SiO_2_, hexane-EtOAc (50:50) furnishing the diol mesylate **15** (0.69 g; 49%) as a pale yellow oil. ^1^H-NMR (300 MHz, CDCl_3_) δ 5.25–5.46 (m, 8H), 4.34–4.38 (m, 2H), 3.75–3.83 (m, 1H), 3.67–3.75 (m, 1H), 3.05 (s, 3H), 2.57–2.85 (m, 6H), 2.11–2.40 (m, 3H), 1.97–2.11 (m, 2H), 1.46–1.71 (m, 3H), 0.95 (t, *J* = 9 Hz, 3H). ^13^C-NMR (75 MHz, CDCl_3_) δ 132.31 (CH), 129.39 (CH), 129.21 (CH), 128.87 (CH), 128.58 (CH), 128.26 (CH), 128.03 (CH), 127.22 (CH), 72.81 (CH), 71.83 (CH), 71.14 (CH), 37.72 (CH_3_), 32.61 (CH_2_), 25.84 (CH_2_), 25.77 (2×CH_2_), 23.68 (CH_2_), 20.78 (CH_2_), 14.49 (CH_3_). IR: 3511, 3011, 1650 cm^−1^. MS (EI) *m/z* (rel. %): 130 (12), 118 (25), 107 (33), 92 (44), 90 (62), 78 (100), 66 (44), 54 (94). HRMS (EI). Calculated for C_19_H_32_O_5_S: 372.1970; Found 372.1990.

*4Z,7Z,10Z,13Z-hexadecatetraenal* (**5**)—Method 1: To a solution of 5% LiOH in MeOH-H_2_O (1:1) (20 mL), acidified with saturated citric acid to pH 4, a solution of the diol mesylate **15** (0.64 g, 1.7 mmol,) in MeOH (2 mL) was added. To the reaction mixture, cooled in ice, NaIO_4_ (0.45 g, 2.1 mmol) was added and the mixture was left stirring at room temperature for 1 h. After saturation with solid NaCl the reaction mixture was extracted with hexane (3 × 25 mL). The extract was washed with water (2 × 25 mL), brine (2 × 25 mL) and dried (MgSO_4_). Evaporation under reduced pressure gave the aldehyde **5** (0.2 g; 50%) as a colorless oil. Spectral data were in agreement with those reported [20].

*4Z,7Z,10Z,13Z-hexadecatetraenal* (**5**)—Method 2: *t*-BuOK (5.5 g, 49 mmol) was added portionwise to an ice-cooled suspension of (methoxymethyl)triphenylphosphonium chloride (17.83 g, 52 mmol) in dry THF (100 mL). After stirring for 15 min at 0 °C, a solution of the aldehyde **6** (5.67 g, 26 mmol) in dry ether (50 mL) was added. The mixture was left stirring at 4 °C overnight. Water (100 mL) was added and volatile compounds were removed under reduced pressure. The reaction mixture was extracted with ether (3 × 50 mL) and the extract was concentrated under reduced pressure. The residue was dissolved in dioxane (225 mL) and cooled to 0 °C. Aq. formic acid (225 mL; 80%) was added and the mixture was left stirring overnight at room temperature. Water (100 mL) was added and volatile compounds were removed under reduced pressure. The reaction mixture was extracted with hexane (3 × 50 mL) and the extract was washed successively with aq. NaHCO_3_ (2 × 50 mL), water (2 × 50 mL) and brine (50 mL) and dried (MgSO_4_). Evaporation of solvents under reduced pressure followed by flash column chromatography (SiO_2_, CH_2_Cl_2_) gave the aldehyde **5** (3.31 g; 38%) as a colorless oil. Spectral data were in agreement with those previously reported [20].

*1,6Z,9Z,12Z,15Z-octadecapentaen-3-ol* (**3**). A solution of the aldehyde **5** (2.31 g, 10 mmol) in dry Et_2_O (20 mL) was added dropwise to a solution of vinyl magnesium bromide in THF (20 mL, 1M, 20 mmol) at 0 °C. The mixture was stirred for 1 h at 0 °C and quenched with saturated aq. NH_4_Cl (30 mL). The product was extracted with Et_2_O (3 × 25 mL) and the extract dried (MgSO_4_). Evaporation under reduced pressure gave a residue that was purified by flash chromatography (SiO_2_, hexane-EtOAc (80:20)) to give the alcohol **3** (4.23 g; 63%) as a colorless oil. ^1^H-NMR (300 MHz, CDCl_3_) δ 5.86 (ddd, *J* = 6 Hz, *J* = 12 Hz, *J* = 18 Hz, 1H), 5.27–5.44 (m, 8H), 5.22 (d, *J* = 18 Hz, 1H), 5.10 (d, *J* = 9 Hz, 1H), 4.11 (q, *J* = 6 Hz, 1H), 2.69–2.88 (m, 6H), 1.99–2.21 (m, 4H), 1.47–1.64 (m, 3H) 0.96 (t, *J* = 6 Hz, 3H). ^13^C-NMR (75 MHz, CDCl_3_) δ 141.29 (CH), 132.29 (CH), 129.66 (CH), 128.79 (CH), 128.74 (CH), 128.48 (CH), 128.41 (CH), 128.15 (CH), 127.26 (CH), 114.99 (CH_2_), 72.93 (CH), 36.99 (CH_2_), 25.87(CH_2_), 25.78 (2×CH_2_), 23.46 (CH_2_), 20.79 (CH_2_), 14.51 (CH_3_). IR: 3347, 3011, 1645 cm^−1^. MS (EI) *m/z* (rel. %): 130 (12), 116 (20), 107(28), 104 (32), 92(40), 90(65), 78(100), 66 (51), 54 (38), 40(52). HRMS (EI). Calculated for C_18_H_28_O: 260.2140; Found 260.2141.

*1,6Z,9Z,12Z,15Z-octadecapentaen-3-one* (**1**). A solution of the alcohol **3** (0.31 g, 1.2 mmol) in CH_2_Cl_2_ (5 mL) was added to a suspension of Dess-Martin periodane (DMP) (0.64 g, 1.5 mmol) in CH_2_Cl_2_ (30 mL) at room temperature. The mixture was stirred for 1 h at room temperature. Saturated aq. KHCO_3_ (30 mL) and 10% saturated aq. Na_2_S_2_O_3_ (30 mL) were added, and the product was extracted with CH_2_Cl_2_ (3 × 10 mL). The extract was washed successively with water (2 × 10 mL) and brine (2 × 10 mL) and dried (MgSO_4_). Evaporation of the solvent under reduced pressure gave a residue that was purified by flash chromatography (SiO_2_, hexane-EtOAc (80:20)) to give **1** (0.27 g; 89%) as a colorless oil. ^1^H-NMR (300 MHz, CDCl_3_) δ 6.36 (dd, *J* = 12 Hz, *J* = 18 Hz, 1H), 6.21 (dd, *J* = 3 Hz, *J* = 18 Hz, 1H*_trans_*), 5.83 (dd, *J* = 3 Hz, *J* = 9 Hz, 1H*_cis_*), 5.24–5.43 (m, 8H), 2.75–2.87 (m, 6H), 2.63 (t, *J* = 9 Hz, 2H), 2.33–2.43 (m, 2H), 2.00–2.17 (m, 2H), 0.95 (t, *J* = 9 Hz, 3H). ^13^C-NMR (75 MHz, CDCl_3_) δ 200.25 (C=O), 136.72 (CH), 132.29 (CH), 129.24 (CH), 128.78 (CH), 128.48 (2×CH), 128.26 (2×CH), 128.05 (CH), 127.21 (CH), 39.56 (CH_2_), 25.82 (CH_2_), 25.78 (CH_2_), 25.74 (CH_2_), 21.92 (CH_2_), 20.75 (CH_2_), 14.46 (CH_3_). IR: 3011, 1702, 1682, 1615 cm^−1^. MS (EI) *m/z* (rel. %): 130 (14), 118 (28), 107 (39), 92 (48), 90 (66), 78 (100), 66 (45), 54 (95). HRMS (EI) Calculated for C_18_H_26_O: 258.1984; Found 258.1995. Spectral data were in accord with those reported for the natural product [2].

## 4. Conclusions

In summary, we have completed the first total synthesis of 1,6*Z*,9*Z*,12*Z*,15*Z*-octadecapentaen-3-one (**1**) in 6 steps and 17% overall yield from EPA ethyl ester. The coupling between the C-16 aldehyde **5** and vinyl magnesium bromide is the key step. The synthesis of vinyl ketone analogues of **1** is currently in progress in our laboratory.
